# Solid Papillary Carcinoma of Breast: Clinicopathologic Comparison With Conventional Ductal Carcinoma of Breast

**DOI:** 10.7759/cureus.11172

**Published:** 2020-10-26

**Authors:** Atif A Hashmi, Syeda N Iftikhar, Rimsha Haider, Raviha Haider, Muhammad Irfan, Javaria Ali

**Affiliations:** 1 Pathology, Liaquat National Hospital and Medical College, Karachi, PAK; 2 Internal Medicine, Liaquat National Hospital and Medical College, Karachi, PAK; 3 Emergency Medicine, National Institute of Blood Diseases and Bone Marrow Transplantation, Karachi, PAK; 4 Internal Medicine, Ziauddin Medical University, Karachi, PAK; 5 Statistics, Liaquat National Hospital and Medical College, Karachi, PAK

**Keywords:** solid papillary carcinoma, papillary breast tumors, breast cancer, invasive ductal carcinoma, synaptophysin, estrogen receptor, progesterone receptor, human epidermal growth factor receptor-2

## Abstract

Introduction

Solid papillary carcinoma (SPC) is a distinct rare subtype of breast tumour that often exhibits a neuroendocrine differentiation. Due to the rarity of these tumours, few studies have assessed the clinicopathological features of these tumours. Therefore, in this study, we evaluated the clinical and pathological profiles of SPC and compared the pathologic features with conventional invasive ductal carcinoma (IDC) in our population.

Methods

It was a retrospective cross-sectional study conducted at Liaquat National Hospital and Medical College from January 2013 until December 2019 over seven years. Cases with histological diagnosis of SPC and IDC were included in the study, and clinicopathological characteristics were compared.

Results

We included 39 cases of SPC in our study diagnosed during the study period. During the same timeline, 634 cases of IDC were reported and therefore included in the study for comparison. The mean age of the patients with SPC was 53.97 ± 12.15 years, and the mean tumour size was 3.42 ± 1.87 cm. Axillary metastasis was noted in 15.4% of cases. 94.9% of cases of SPC were invasive. Estrogen receptor (ER), progesterone receptor (PR), human epidermal growth factor receptor-2 (HER2/neu) and synaptophysin positivity was seen in 84.6%, 87.2%, 10.3%, and 59% respectively. Recurrence was noted in 10.3% of cases with 94.9% survival rate. Cases of SPC had significantly lower grade (grade I + II), tumour (T) and nodal (N) stage than IDC. Moreover, the frequency of hormonal receptor expression (ER and PR) was higher, and the frequency of human epidermal growth factor receptor 2 (HER2/neu) expression was lower compared to IDC.

Conclusion

SPC is a distinct variant of malignant papillary breast tumours with overall better prognostic parameters than IDC. Therefore, it is essential to recognize the histological features of this rare breast tumour.

## Introduction

In Southeast Asia, breast cancers are common and typically present late and are associated with adverse prognostic parameters [1,2]. Invasive ductal carcinoma (IDC) of the breast is the most common histologic subtype of breast cancer with a distinct pathological and prognostic profile [3,4]. Papillary breast neoplasms are a heterogeneous group of tumours, ranging from benign intraductal papilloma to malignant invasive papillary carcinoma [5]. Between these two extremes, there lie categories of in situ papillary carcinoma. Two notoriously deceptive in situ tumours in this category include solid papillary carcinoma (SPC) and encapsulated papillary carcinoma. SPC is further divided into SPC in situ and SPC invasive based on the presence or absence of invasive component. Although SPC in situ mostly lacks a myoepithelial layer at the periphery of the tumour, they are still considered in situ tumours. Histologically, SPC is characterized by multiple circumscribed nodules of a tumour with a smooth outline [6]. They often show neuroendocrine differentiation, characterized by synaptophysin positivity and sometimes also exhibit mucinous component. The invasive component in SPC can be in the form of conventional invasive ductal carcinoma (IDC) or invasive carcinoma with SPC like features. The invasive component derives the tumour stage (T-stage). Due to the rarity of these tumours, few studies have assessed the clinicopathological features of these tumours. Therefore, in this study, we evaluated the clinical and pathological profiles of SPC and compared the pathologic features with conventional IDC in our population.

## Materials and methods

**Figure 1:**
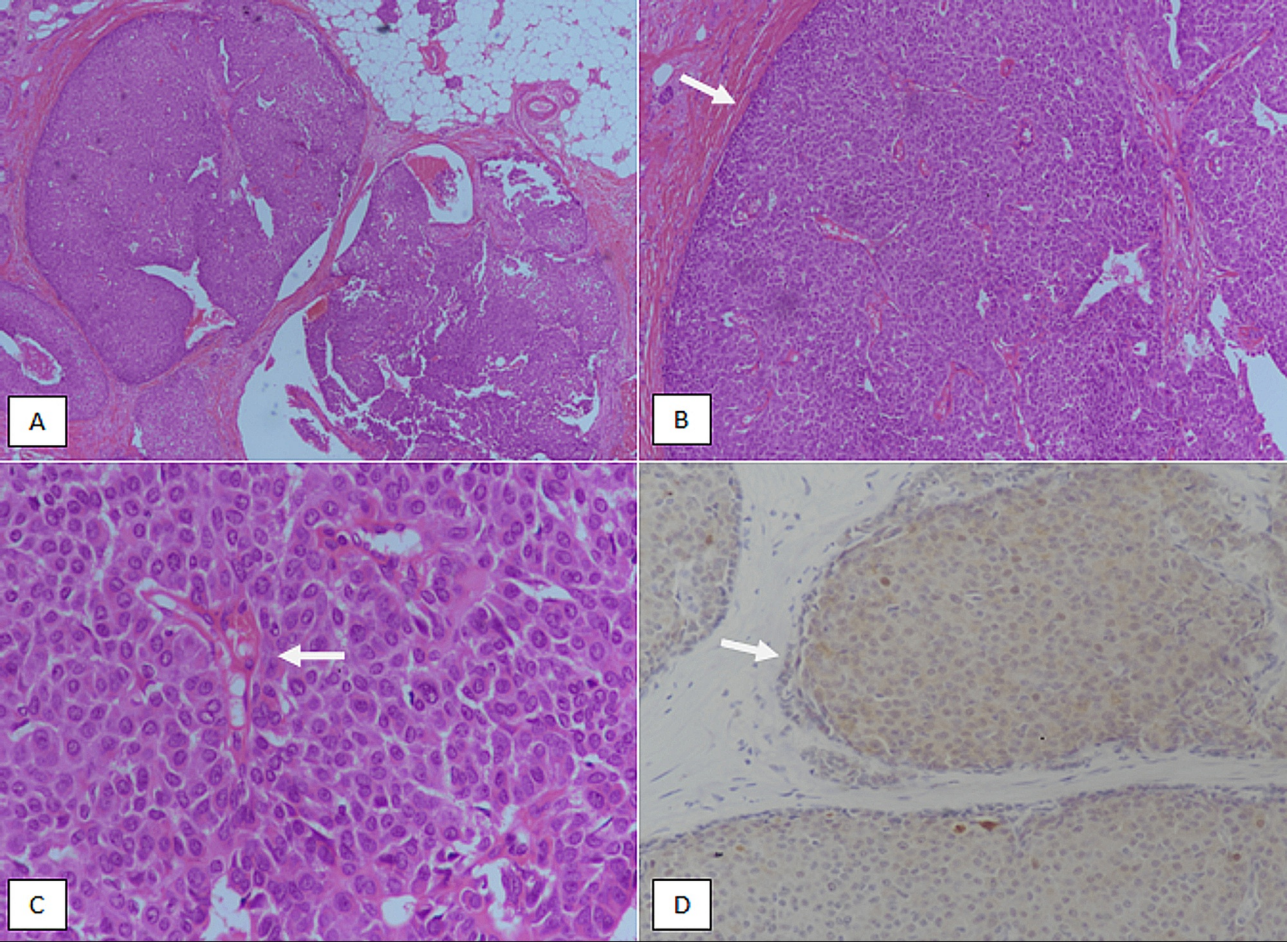
Solid papillary carcinoma in situ. (A): H & E sections at 40X magnification showing discrete nodules of tumor. (B): 100X magnification showing circumscribed borders (arrow). (C): 200X magnification showing papillary cores (arrow) and low-grade nuclear atypia. (D): p63 immunostain showing lack of nuclear myoepithelial staining at the periphery of the tumor nodules. H & E, Hematoxylin and eosin

**Figure 2:**
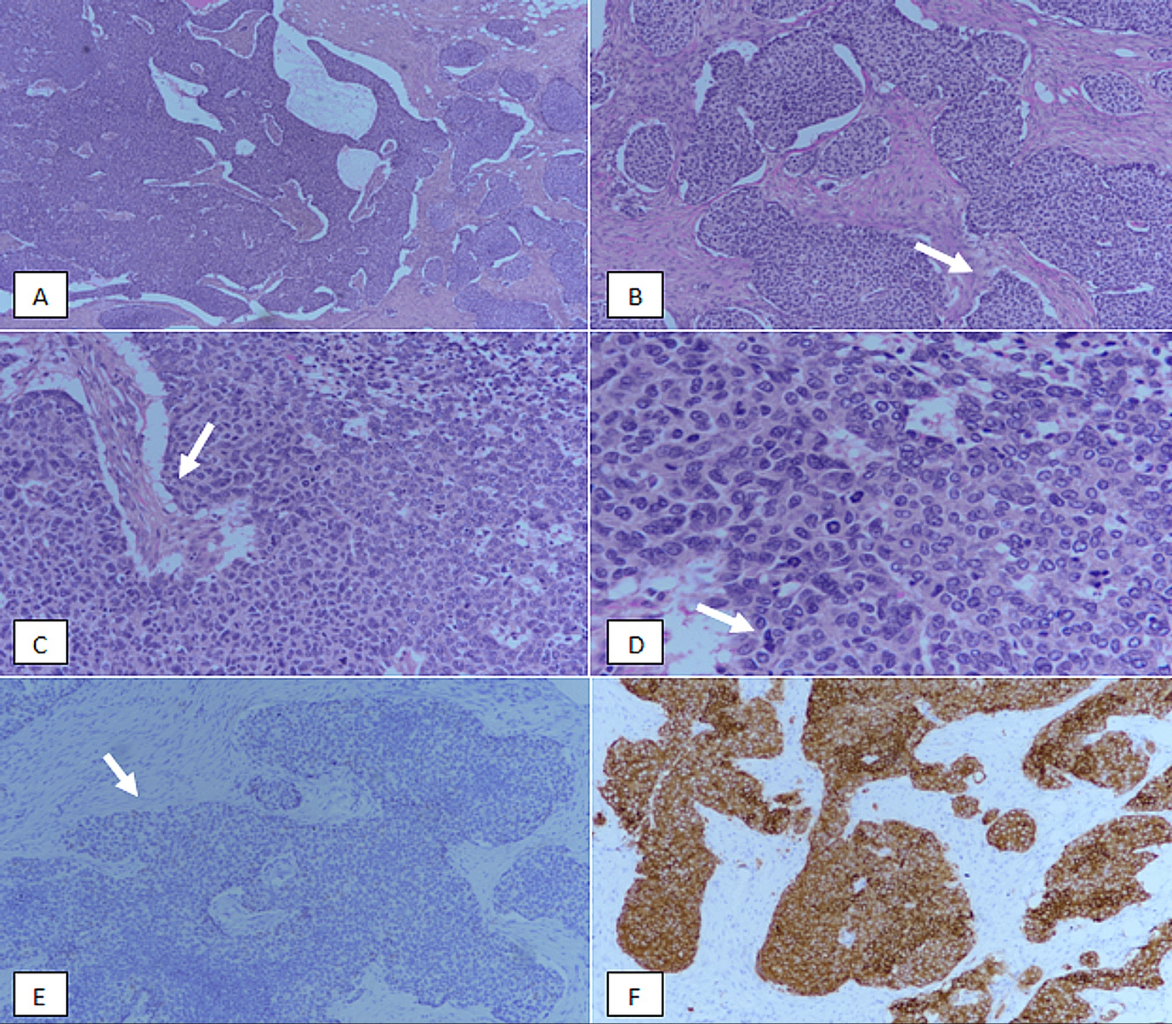
Solid papillary carcinoma invasive. (A): H & E-stained sections at 40X magnification showing multiple nodules of a tumour. (B): 100X magnification showing irregular borders of the tumour (arrow). (C): 200X magnification revealing fibrovascular cores (arrow). (D): 400X magnification showing high-grade nuclear atypia with mitosis (arrow). (E): p63 immunostain showing lack of nuclear myoepithelial staining at the periphery of the tumour foci (arrow). (F): Synaptophysin immunostain revealing diffuse strong positivity in tumour cells. H & E, Hematoxylin and eosin

It was a retrospective cross-sectional study conducted at Liaquat National Hospital and Medical College from January 2013 until December 2019 over seven years. All patients underwent resections of breast tumours after biopsy diagnosis of atypical papillary breast tumours. Specimens included a lumpectomy, simple mastectomy with sentinel lymph node dissection and modified radical mastectomy (MRM). Intra-operative frozen section for sentinel lymph nodes was performed for patients with clinically and radiologically negative axillary lymph nodes. In cases where the operative plan was mastectomy, any positive sentinel lymph node (macrometastasis) on the frozen section was followed by axillary lymph node dissection. Alternatively, for patients undergoing breast conservation surgery, a minimum of three positive sentinel lymph nodes on the frozen section with at least one macrometastasis was followed by axillary dissection. The axillary dissection without prior sentinel lymph node biopsy was performed in cases with clinically or radiologically positive lymph nodes after trucut biopsy/fine needle aspiration cytology confirmation of malignancy. Gross examination and sampling of tumours were done according to standard protocols. Hematoxylin and eosin-stained sections were first examined, and then myoepithelial stains were applied on representative tissue blocks. Cases with histological diagnosis of SPC and IDC were included in the study, and clinicopathological characteristics were compared. Estrogen receptor (ER), progesterone receptor (PR) and human epidermal growth factor receptor-2 (HER2/neu) immunohistochemistry (IHC) was applied to all cases of SPC and IDC. Cases with equivocal (2+) IHC for HER2/neu were further tested by fluorescence in situ hybridization (FISH). Cases with SPC, there were two cases with equivocal HER2/neu IHC (2+), and both were FISH negative. For IDC, 64 cases were equivocal (2+) for HER2/neu IHC, out of which 38 cases revealed HER2/neu amplification on FISH and therefore labelled as Her2/neu positive on the final analysis. The rest of the cases (non-amplified) were taken as HER2/neu negative. Also, synaptophysin IHC was performed on all cases of SPC. Histological images and myoepithelial stains of in situ and invasive SPC are shown in Figures 1, 2, respectively.

Data analysis was performed using Statistical Package for Social Sciences (Version 26.0, IBM Inc., Armonk, USA). Chi-square, Fisher exact test and independent t-test were used to check the association. Survival analysis was performed by the Kaplan-Meier method. p-values ≤ 0.05 were considered as significant.

## Results

Clinicopathological features of solid papillary carcinoma

We included 39 cases of SPC in our study diagnosed during the study period. During the same timeline, 634 cases of IDC were reported and therefore included in the study for comparison. The mean age of the patients of SPC was 53.97 ± 12.15 years, and the mean tumour size was 3.42 ± 1.87. Majority of the specimens were lumpectomy (66.7%), and most of the tumours were grade II (74.4%). Axillary metastasis was noted in 15.4% of cases. 94.9% of cases of SPC were invasive. ER, PR, HER2/neu and synaptophysin positivity was seen in 84.6%, 87.2%, 10.3%, and 59% respectively. Median follow-up time for SPC cases was 31 months. Recurrence was noted in 10.3% of cases with 94.9% survival rate. Detailed clinicopathological features of SPC cases under study are presented in Table 1.

**Table 1:** Clinicopathologic characteristics of Solid papillary carcinoma SD, standard deviation; MRM, modified radical mastectomy; T, tumour; N, nodal; Tis, tumor in situ; ER, estrogen receptor; PR, progesterone receptor; HER2/neu, human epidermal growth factor receptor-2; SPC, solid papillary carcinoma

Clinicopathologic characteristic	Frequency (%)
Age (years)	
Mean±SD	53.97±12.15
Age groups	
≤50 years	19(48.7)
>50 years	20(51.3)
Tumor size (cm)	
Mean±SD	3.42±1.87
Tumor size groups	
<2 cm	4(10.3)
2-5 cm	29(74.4)
>5 cm	6(15.4)
Follow up (months) Mean±SD (median)	30.66±10.49 (31)
Specimen type	
Lumpectomy	26(66.7)
Simple mastectomy	3(7.7)
MRM	10(25.6)
N stage	
N0	33(84.6)
N1	3(7.7)
N2	3(7.7)
N3	0(0)
T stage	
Tis	2(5.1)
T1	6(15.4)
T2	26(66.7)
T3	5(12.8)
Grade	
Grade-I	7(17.9)
Grade-II	29(74.4)
Grade-III	3(7.7)
Lymphovascular invasion	
Present	5(12.8)
Absent	34(87.2)
Axillary metastasis	
Present	6(15.4)
Absent	33(84.6)
ER	
Positive	33(84.6)
Negative	6(15.4)
PR	
Positive	34(87.2)
Negative	5(12.8)
HER2/neu	
Positive	4(10.3)
Negative	35(89.7)
Synaptophysin	
Positive	23(59)
Negative	16(41)
Type of SPC	
SPC in situ	2(5.1)
SPC invasive	37(94.9)
Recurrence	
Yes	4(10.3)
No	35(89.7)
Survival status	
Alive	37(94.9)
Expired	2(5.1)

Comparison of solid papillary carcinoma with invasive ductal carcinoma

Table 2 compares the clinicopathologic features of SPC with IDC. Cases with SPC were found significantly to have a lower grade (grade I + II), tumor (T) and nodal (N) stage compared to IDC. Moreover, frequency of hormonal receptor expression (ER and PR) was higher and frequency of HER2/neu expression was lower compared to IDC. However, no significant association was noted with respect to age, tumor size and lymphovascular invasion.

**Table 2:** Comparison of clinicopathologic characteristics of solid papillary carcinoma with invasive ductal carcinoma of breast *Chi-square test was applied, **Fisher Exact test was applied, ***Independent t-test was applied. SD, standard deviation; N, nodal; T, tumor; Tis, tumor in situ; ER, estrogen receptor; PR, progesterone receptor; HER2/neu, human epidermal growth factor receptor-2

Clinicopathologic characteristics	Solid Papillary carcinoma (n=39)	Invasive ductal carcinoma (n=634)	P-value
Age (years)			
Mean±SD	53.97±12.15	51.95±12.15	0.318***
Age groups			
≤50 years	19(48.7)	306(48.3)	0.956*
>50 years	20(51.3)	328(51.7)	
Tumor size (cm)			
Mean±SD	3.42±1.87	3.61±1.48	0.444***
N stage			
N0	33(84.6)	320(50.5)	<0.0001*
N1	3(7.7)	130(20.5)	
N2	3(7.7)	85(13.4)	
N3	0(0)	99(15.6)	
T stage			
Tis	2(5.1)	0(0)	0.007**
T1	6(15.4)	83(13.1)	
T2	26(66.7)	454(71.6)	
T3	5(12.8)	97(15.3)	
Grade			
Grade-I	7(17.9)	53(8.4)	<0.0001**
Grade-II	29(74.4)	293(46.2)	
Grade-III	3(7.7)	288(45.4)	
Lymphovascular invasion			
Present	5(12.8)	157(24.8)	0.09*
Absent	34(87.2)	477(75.2)	
Axillary metastasis			
Present	6(15.4)	314(49.5)	<0.0001*
Absent	33(84.6)	320(50.5)	
ER			
Positive	33(84.6)	399(62.9)	0.006*
Negative	6(15.4)	235(37.1)	
PR			
Positive	34(87.2)	323(50.9)	<0.0001*
Negative	5(12.8)	311(49.1)	
HER2/neu			
Positive	4(10.3)	223(35.2)	0.001*
Negative	35(89.7)	411(64.8)	

Survival analysis of solid papillary carcinoma for tumour grade and size

**Figure 3:**
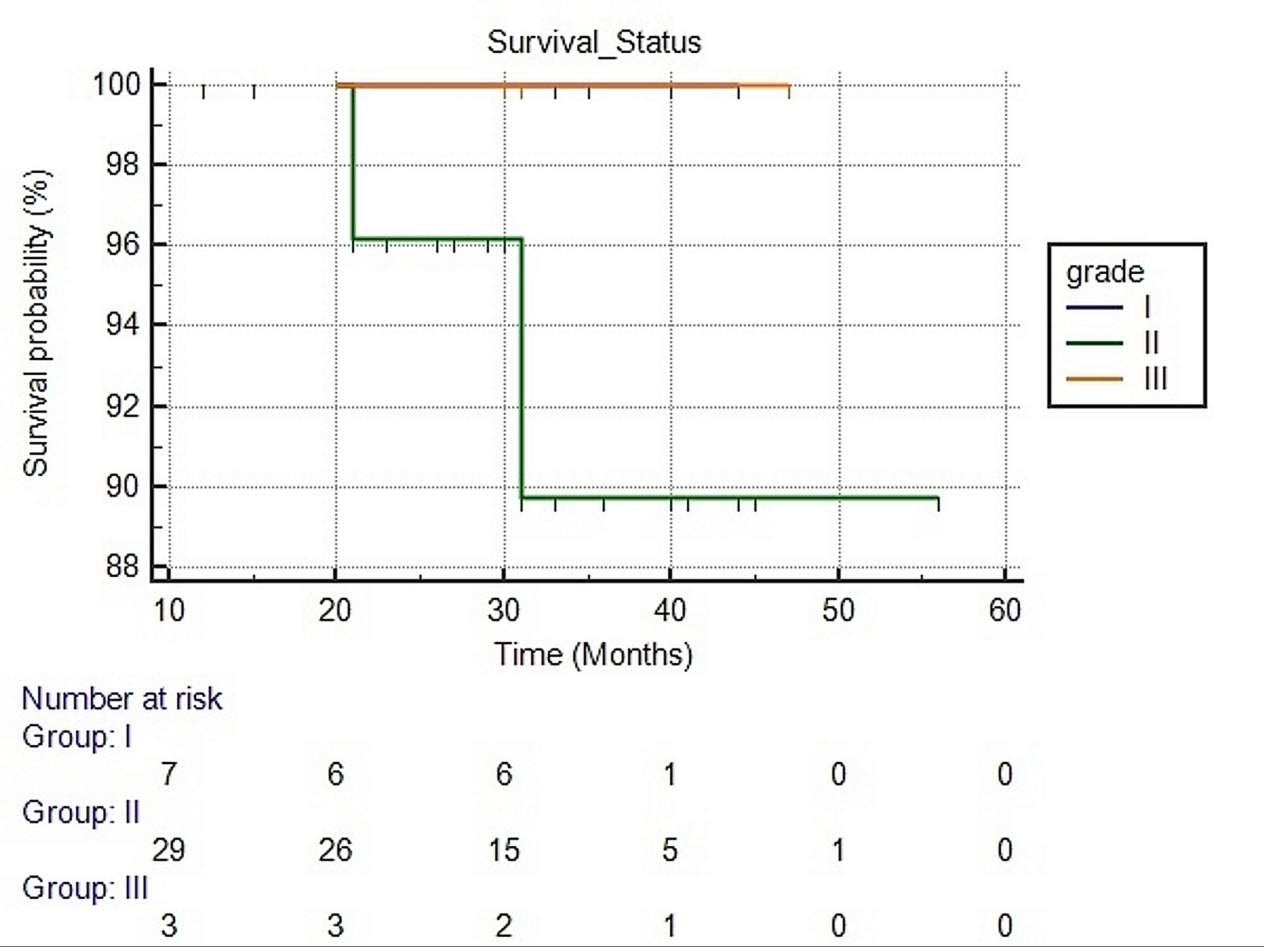
Survival analysis using the Kaplan-Meier method for Solid papillary carcinoma with respect to tumor grade

**Figure 4:**
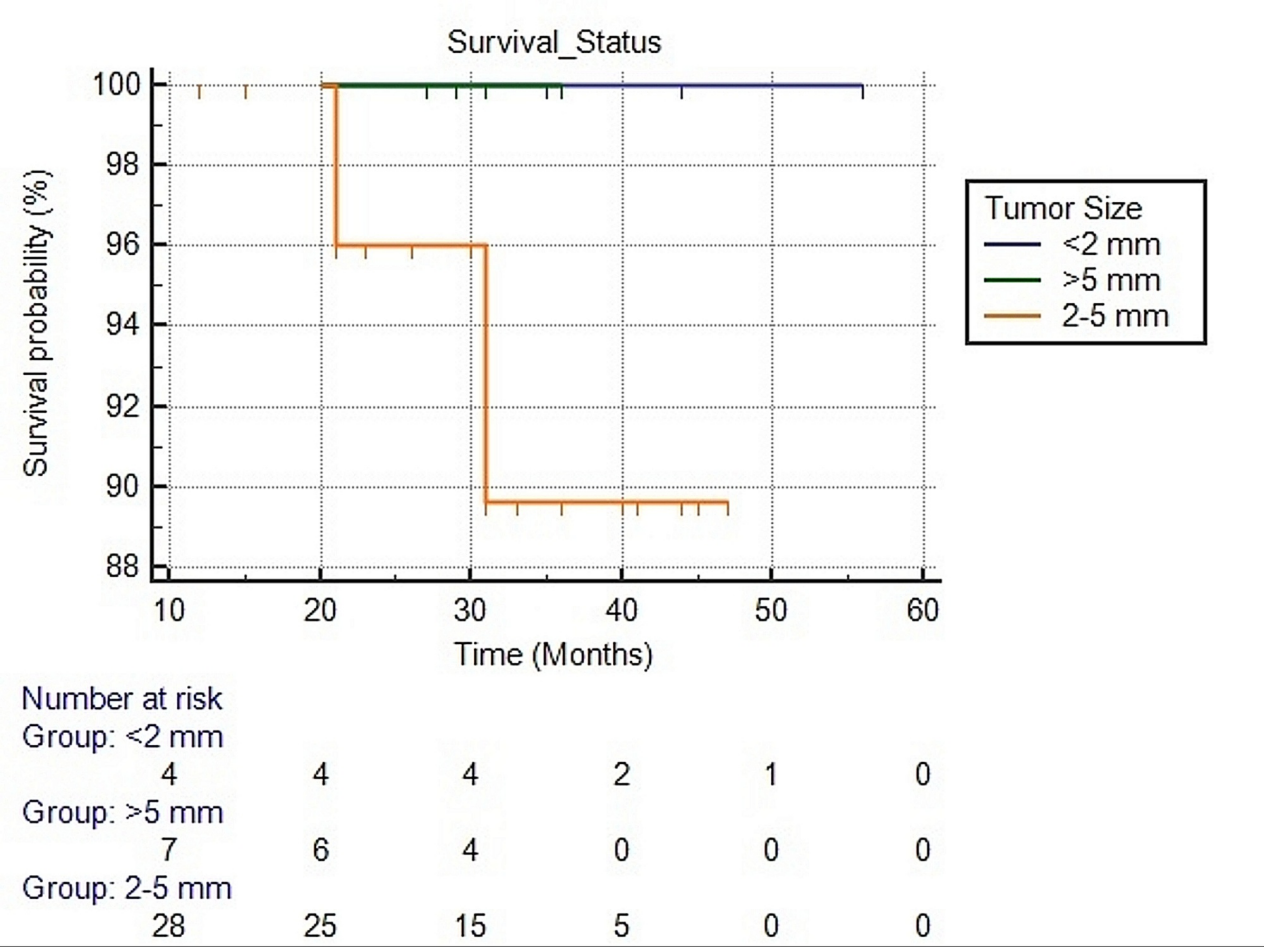
Survival analysis using the Kaplan-Meier method for Solid papillary carcinoma with respect to tumor size

We evaluated the survival status of SPC cases with respect to tumour grade and size using Kaplan-Meier curves; however, no significant difference in survival was seen with p-values (log-rank) of 0.645 and 0.628, respectively as presented in Figures 3, 4.

## Discussion

In this study, we evaluated the clinicopathological features of SPC and compared with IDC diagnosed during the same study period. We found that SPC had overall better prognostic features than IDC as cases of SPC were found to have a lower grade, T-stage and N-stage than IDC. Moreover, the overall survival of SPC cases was over 90% with a low frequency of recurrence in our study.

SPC is a distinct type of breast carcinoma that is staged as in situ carcinoma unless associated with invasive cancer or exhibit jagged infiltrative borders. A study conducted in Singapore evaluated the clinicopathologic characteristics of SPC. The authors concluded that invasive carcinoma associated with SPC was of small size and low grade [7]. In our study, more than 90% of SPC cases had an invasive component, and most were low grade (I and II).

SPC is considered an indolent variant of breast carcinoma. A study involving 20 cases of SPC did not reveal nodal metastasis in any case [8]. In our study, nodal metastasis was seen in 15.4% of cases, which is less compared to IDC (49.5%). Another unique feature of SPC is a neuroendocrine differentiation. A study revealed that 45% of cases of SPC exhibited neuroendocrine differentiation [9]. In our series of cases, synaptophysin positivity was seen in 59% cases. It is essential to differentiate SPC from IDC with neuroendocrine differentiation that lacks the typical nodular pattern and papillary cores characteristic of SPC.

We acknowledge a few limitations to our study, the most important of which is the small sample size. Second, the follow-up for cases of IDC was not available for comparison with SPC. Therefore, we recommend extensive prospective cohort studies to evaluate the prognosis of SPC in our population with a comparison of survival with IDC.

## Conclusions

SPC is a distinct malignant papillary tumour of the breast with neuroendocrine differentiation and good overall survival. Pathologically, SPC possesses better prognostic parameters such as lower tumour grade and TN-stage than IDC. Histologically, SPC is characterized by discrete nodules of tumour cells with low-grade nuclei and circumscribed borders; however, it is of utmost importance to recognize the invasion as we found invasion in a high percentage of cases in our study. Like other papillary breast lesions, the role of myoepithelial stains is of crucial importance to differentiate SPC from papillary DCIS. In addition, synaptophysin immunostaining also have a diagnostic role in SPC as we found its positivity in a substantial number of cases of SPC. Therefore, it is essential to recognize histological and immunohistochemical characteristics of SPC, especially on trucut biopsy to prognostically stratify patients with breast cancer.
